# Over-Production of Physicians

**Published:** 1898-09

**Authors:** 


### OVER-PRODUCTION OF PHYSICIANS.

The following notice, taken from a clipping in the *American Journal of Surgery and Gynecology*, is certainly well worth the reading. It shows that the same condition of affairs in the United States has its counterpart in other countries. What will be the sequel time alone can tell.

"Our Canadian brethren seem to be as much troubled with the over-production of doctors as are we of the States, judging from what the *Canadian Journal of Medicine and Surgery* remarks: "That in some walks of life the struggle for an existence should be so strenuous is, we think, a pretty sure indication that these vocations are not only overcrowded, but overcrowded to the danger point. Wherever human energy is concentrated there will necessarily be competition, and to the spirit of competition or peaceful rivalry is perhaps due every step of human progress. But of late years another meaning has been given to the word competition which differs very widely from the original signification. It is natural and paiseworthy that a worker in any capacity should be ambitious to do better work than his fellows. It is right that a man should be fond of his work and proud of it when he attains to excellence in it; and when members of the same craft or the same profession vie with one another in the performance of the most perfect workmanship, as much for love of their art as for the material guerdon which may ensue, they may be said to be in legitimate competition with one another; and without such competition life would be indescribably flat. But when the amount of work to be done is limited and those equipped to perform it are greatly in excess of the number needed for its accomplishment, it must necessarily follow that only a few will have anything to do at all and that the large majority will be unemployed. To this condition of things the term competition has been, we think, falsely applied of late years.

The labor to be accomplished is viewed in the light of spoils, and the horde of workmen surround the coveted prey in turbulent warfare with one another. Thrice happy is he who in this war to the knife is able to snatch from the midst of the conflict some portion of the plunder and bear it away. However, it has been said that all is fair in love or war. In this war of competition a great many methods seem to be regarded as fair which, without the light of our venerable proverb, would hardly be so regarded. Peculiarly to the present conditions in the medical profession do these remarks apply, for of all branches of handicraft the physician's seems to be the most overcrowded. The great desideratum of the present day seems to be a 'respectable' calling. We suppose this is one of the unfortunate indications of the prevailing vulgarity of the time. One of the first results of university extension and universal education of all classes will be that everybody will wish to live by his wits, or, as it is sometimes more pompously expressed, be a 'brain-worker,' forgetful of the fact that we all cannot be parasites! The world will support only a limited number of parasites, whether they be physicians, barristers, clergymen or artists; as a tree in the South will support only a given weight of the Spanish moss. When the moss thrives beyond the tree's power of support, it kills the tree, and with the fall of the tree comes the extermination of the vegetable parasite. All cannot be doctors and ministers and musicians, but a great part of the population, unfortunately, has set up as one or the other. This unnatural condition will last until it brings its own heavy punishment, and then will come the slow and painful readjustment. At present, however, the parasitical class seem in a fair way to destroy the supporting class, and the quarreling among the parasites affords an amusing tragi-comedy for the cynical observer."

